# Benefits of Prophylactic Short-Course Immune Tolerance Induction in Patients With Infantile Pompe Disease: Demonstration of Long-Term Safety and Efficacy in an Expanded Cohort

**DOI:** 10.3389/fimmu.2020.01727

**Published:** 2020-08-06

**Authors:** Ankit K. Desai, Carolyn H. Baloh, John W. Sleasman, Amy S. Rosenberg, Priya S. Kishnani

**Affiliations:** ^1^Division of Medical Genetics, Department of Pediatrics, Duke University Health System, Durham, NC, United States; ^2^Division of Allergy, Immunology, and Pulmonary Medicine, Department of Pediatrics, Duke University Health System, Durham, NC, United States; ^3^Division of Biologics Review and Research 3, Office of Biotechnology Products, Center for Drug Evaluation and Research, US FDA, Bethesda, MD, United States

**Keywords:** anti-drug antibodies, enzyme replacement therapy, immune tolerance induction, anti-rhGAA IgG antibody, alglucosidase alfa, immunogenicity

## Abstract

Immune tolerance induction (ITI) with a short-course of rituximab, methotrexate, and/or IVIG in the enzyme replacement therapy (ERT)-naïve setting has prolonged survival and improved clinical outcomes in patients with infantile Pompe disease (IPD) lacking endogenous acid-alpha glucosidase (GAA), known as cross-reactive immunologic material (CRIM)-negative. In the context of cancer therapy, rituximab administration results in sustained B-cell depletion in 83% of patients for up to 26–39 weeks with B-cell reconstitution beginning at approximately 26 weeks post-treatment. The impact of rituximab on serum immunoglobulin levels is not well studied, available data suggest that rituximab can cause persistently low immunoglobulin levels and adversely impact vaccine responses. Data on a cohort of IPD patients who received a short-course of ITI with rituximab, methotrexate, and IVIG in the ERT-naïve setting and had ≥6 months of follow-up were retrospectively studied. B-cell quantitation, ANC, AST, ALT, immunization history, and vaccine titers after B-cell reconstitution were reviewed. Data were collected for 34 IPD patients (25 CRIM-negative and 9 CRIM-positive) with a median age at ERT initiation of 3.5 months (0.1–11.0 months). B-cell reconstitution, as measured by normalization of CD19%, was seen in all patients (*n* = 33) at a median time of 17 weeks range (11–55 weeks) post-rituximab. All maintained normal CD19% with the longest follow-up being 248 weeks post-rituximab. 30/34 (88%) maintained negative/low anti-rhGAA antibody titers, even with complete B-cell reconstitution. Infections during immunosuppression were reported in five CRIM-negative IPD patients, all resolved satisfactorily on antibiotics. There were no serious sequelae or deaths. Of the 31 evaluable patients, 27 were up to date on age-appropriate immunizations. Vaccine titers were available for 12 patients after B-cell reconstitution and adequate humoral response was observed in all except an inadequate response to the Pneumococcal vaccine (*n* = 2). These data show the benefits of short-course prophylactic ITI in IPD both in terms of safety and efficacy. Data presented here are from the youngest cohort of patients treated with rituximab and expands the evidence of its safety in the pediatric population.

## Introduction

Lysosomal storage disorders (LSDs) are a group of inherited metabolic disorders caused by disease-associated variants in genes encoding catabolic enzymes active in the lysosome. The deficiency or complete absence of endogenous enzyme leads to a build-up of undegraded macromolecules in lysosomes affecting various target tissues depending on the specific enzyme deficiency (1–3). Although there is no cure for LSDs, the development of enzyme replacement therapy (ERT) aimed at replacing the deficient lysosomal enzymes and reducing the toxic substrate accumulation has greatly improved the course for several LSDs including Gaucher disease, Fabry disease, Pompe disease, Mucopolysaccharidoses (MPS) I, II, IVA, VI, and VII, and Wolman disease, with additional therapeutic proteins in development (4, 5). Despite the success of ERTs in improving outcomes for patients with LSDs, the development of anti-drug antibodies (ADA) against the therapeutic protein remains a challenge that impacts both the safety and efficacy of the treatment (6).

In 2006, the FDA approved alglucosidase alfa (recombinant human acid alfa-glucosidase, rhGAA) for treatment of Pompe disease, an autosomal recessive LSD caused by disease-associated variants in the *GAA* gene resulting in deficiency of acid-alpha glucosidase (GAA), predominantly affecting skeletal, smooth, and cardiac muscle (7, 8). Pompe disease has a phenotypic spectrum ranging from classic infantile Pompe disease (IPD) to late-onset Pompe disease. Classic IPD is the most severe end of the disease spectrum, with patients presenting with severe cardiomyopathy in the first few days to weeks of life and rarely surviving beyond 2 years of age without treatment (9). The availability of ERT with alglucosidase alfa has changed the natural course of Pompe disease, significantly prolonging survival and improving long-term clinical outcomes. Despite the considerable benefits of ERT, the overall response is heterogeneous and impacted by multiple factors including age at ERT, the extent of underlying pathology, the dose of ERT, and development of anti-drug antibodies. Additionally, there are other limitations of ERT including clearance by non-muscle tissue, limited cellular uptake in muscles, inability to cross blood-brain barrier, and variability of skeletal muscle response (10, 11). Published literature has demonstrated that long-term IPD survivors often have residual physical impairments including muscle weakness, hypernasal speech, dysphagia with a risk of aspiration, ptosis, and risk of arrhythmias (12).

The negative impact of IgG ADA to alglucosidase alfa in patients with IPD has been well established since the first clinical trial (13–15). In two alglucosidase alfa clinical trials, 89% (35/39) of patients (NCT00125879, *n* = 16/18 and NCT00053573, *n* = 19/21) with IPD developed ADA to alglucosidase alfa and a subset developed high and sustained IgG antibody titers (HSAT) causing suboptimal treatment response resulting in clinical deterioration and death despite treatment with ERT (9, 14). Of critical importance, the development of ADA to ERT is strongly influenced by the patient's genetic variants which determine whether any GAA protein is generated, even if non-functional, as the production of a non-functional enzyme may still tolerize the immune system to some extent. Patients with two null variants, produce no GAA, resulting in the immune system recognizing rhGAA as foreign (16). IPD patients with two null *GAA* variants are considered cross-reactive immunologic material (CRIM)-negative and are at the highest risk of developing significant ADA to ERT (16, 17). A previous study assessing the impact of CRIM status on treatment outcomes in Pompe disease showed that CRIM-negative patients who received ERT monotherapy were either deceased or ventilator-dependent by age of 27.1 months due to the development of ADA (16). Immune tolerance induction (ITI) in the ERT-naïve setting has been established as a strategy to diminish the development and minimize the impact of ADA on treatment response to ERT and has become the standard of care for CRIM-negative IPD patients (18–23). Although endogenous GAA detected in CRIM-positive IPD patients can tolerize them to ERT, up to 32% of CRIM-positive IPD patients also develop deleterious ADA to ERT and the clinical course is indistinguishable from that of CRIM-negative IPD patients (24). Some CRIM-positive IPD patients at high risk of developing deleterious ADA can be identified based on previously reported *GAA* variants in IPD patients who developed HSAT or based on development of HSAT by an older sibling. We thus have instituted an immunomodulation approach at ERT initiation based on an algorithm of early immune response data in this subset of CRIM-positive IPD patients considered as high risk (22–24).

Different approaches to overcoming the challenges of ADA to ERT have been tried in patients with Pompe disease with varying degrees of success (25). These immunomodulation approaches, initiated with ERT, are intended to target B and T cells to induce long-term immune tolerance and improve treatment response to ERT (21, 23, 26, 27). While approaches have differed in terms of drug combinations, all approaches included rituximab, an anti-CD20 monoclonal antibody, which targets antibody-producing B cells. We previously reported success in inducing immune tolerance with a short-course of rituximab, low-dose of methotrexate, with/without IVIG in an international cohort of 19 CRIM-negative IPD patients (23). Combination therapy targeting different cells of the immune system (B and T cells) to prevent the cascade for antibody development was the rationale for using rituximab and methotrexate. Rituximab is a chimeric monoclonal antibody that has been approved for multiple types of malignancy and autoimmune diseases. Methotrexate, an inhibitor of dihydrofolate reductase, impacts rapidly dividing T and B cells (28, 29). Experience from rheumatologic disorders has shown that the addition of methotrexate with other biological therapy has prevented the development of ADA against therapeutic proteins (30). We added IVIG at a dose of 500 mg/kg to provide passive immunity until B cell reconstitution would assure production of antibodies to pathogens. The duration of B cell depletion varied based upon patient age, treatment indication, additional immunosuppressive medication administration, and duration of treatment. An example of this is that children typically achieve B cell reconstitution within 1 year of rituximab discontinuation, while adults can take up to 2 years (31). Long-term follow-up studies show that there is skewing of B cell populations to naïve phenotypes resulting in prolonged low immunoglobulin levels and impaired responses to vaccines (32–37). Published literature of rituximab use in autoimmunity and malignancy has shown that though rare, infections can occur when B cells are undetectable (36, 38, 39).

The short 5-week course of ITI was able to tolerize IPD patients to ERT, as evidenced by negative/low anti-rhGAA IgG antibody titers even after B cell reconstitution and ability to receive age-appropriate routine vaccination. The data suggested that combination therapy was safely tolerated and was successful in inducing immune tolerance in most IPD patients. However, the long-term safety of this approach was not evaluated and published data on the safety of immunomodulation with rituximab, especially in pediatric populations, is limited. At this time, there are limited data in the literature on the long-term safety of rituximab, especially in patients younger than a year of age. Considering that the majority of patients with infantile Pompe disease are initiated on treatment within weeks of birth, it is important to understand the long-term safety of treatment in such a young and medically fragile population. The purpose of this study was to determine if patients with IPD who received rituximab experienced long-term impairment of the immune system, as described in the literature for its use in diverse disease settings. In the current study, we present the long-term safety and efficacy of short-course immune tolerance induction (ITI) in a relatively large cohort of CRIM-negative and CRIM-positive IPD patients by evaluating anti-rhGAA IgG antibody titers, absolute neutrophil count (ANC), aspartate aminotransferase (AST), alanine aminotransferase (ALT), B cell and T cell quantitation, vaccination history and titers against vaccines, left ventricular mass index (LVMI), and overall and ventilator-free survival.

## Materials and Methods

### Patients and Inclusion Criteria

Inclusion criteria for the present study were based on the following; (1) a confirmed diagnosis of IPD with two disease-associated *GAA* variants and low GAA enzyme activity (8), (2) a history of ITI with rituximab, methotrexate, with or without IVIG in ERT-naïve setting, and (3) at least 6 months of follow-up data since initiation of ITI. A retrospective chart review of qualifying patients with IPD was conducted. Data on patients included in the previous publication were reviewed and further longitudinal data since the last publication on patients who met the inclusion criteria were included for the analysis (23).

### Ethics Approval

All patients were enrolled in a Duke institutional review board (IRB)-approved study protocol (Pro00001562; Determination of Cross-Reactive Immunological Material [CRIM] Status and Longitudinal Follow-up of Individuals with Pompe disease; LDN6709 Site 206; ClinicalTrials.gov NCT01665326). One CRIM-negative IPD patient included in this study had IRB/ethics committee approval from a local institution. All other patients were enrolled in the Duke IRB-approved study through written informed consent from a parent or legal guardian (15, 22, 40–42).

### Immune Tolerance Induction (ITI)

The 5-week short-course immune tolerance induction approach included four doses of weekly rituximab (375 mg/m^2^, intravenously), and three cycles of low-dose methotrexate (0.4 mg/kg; three doses per cycle with first three ERT infusions, subcutaneously or orally), as described previously (Supplementary Figure 1) (22). To provide passive immunity during B cell suppression, monthly IVIG at 500 mg/kg was added to the combination therapy during the time of B cell suppression. To avoid any significant interference of IVIG with antibody-dependent cellular cytotoxicity mediated B cell depletion, the first dose of IVIG was administered 24–48 h after the first dose of rituximab. There was no recommended supplier for rituximab, methotrexate, and IVIG in ITI protocol that was shared with treating physicians. To assess the safety of ITI, patients were monitored for incidence of infection around the time of ITI, decrease in ANC, and increase in AST, and/or ALT levels. An ANC of <750 cells/mm^3^ or AST and/or ALT >3 times their respective baseline values were considered as adverse events. Routine vaccinations, except for the flu shot were withheld while patients were B cell suppressed. Vaccinations were resumed following evidence of B cell reconstitution, defined as normalization of CD19% (40).

Patients were classified into three groups based on anti-rhGAA IgG antibody titers; (1) HSAT, defined as titers of ≥51,200 on two or more occasions at or beyond 6 months on ERT (15), (2) sustained intermediate titer (SIT), defined as titers of ≥12,800 and <51,200 on ERT (Lumizyme^TM^ prescribing information) (15, 41), and (3) low titer (LT), defined as titers of ≤6,400. The cutoffs of 51,200 and 12,800 were utilized based on the findings from previous publications and Lumizyme^TM^ prescribing information (15, 41). Previous studies have demonstrated that CRIM-positive and CRIM-negative IPD patients who developed anti-rhGAA IgG titers of ≥12,800 had poor clinical outcomes (15, 17, 42). Additionally, the Lumizyme prescribing information states that patients developing sustained anti-alglucosidase alfa antibody titers of ≥12,800 may have a poorer clinical response to treatment, or may lose motor function as antibody titers increase. Patients with antibody titers ≥12,800 at Week 12 of treatment had an average increase in alglucosidase alfa clearance of 50% from Week 1 to 12. In our study with ITI, patients were considered immune tolerant if they met the following criteria: (1) were seronegative (did not develop anti-rhGAA IgG antibodies) or maintained anti-rhGAA IgG titers of ≤6,400 throughout ERT, and (2) were able to receive age-appropriate routine vaccines.

### Data Collection

Clinical data including *GAA* variants, CRIM status, age at diagnosis, age at ERT initiation, dose of ERT, longitudinal anti-rhGAA IgG antibody titers, LVMI, motor status, feeding status, and pulmonary status were extracted from medical records provided by the principal care provider of the patient. CRIM status was determined by western blot analysis in skin or blood at Duke GSD/LSD Enzymology Laboratory and confirmed by *GAA* variants or was predicted based on *GAA* variants as previously described (43). Anti-rhGAA IgG antibody titers were determined by Sanofi Genzyme (Framingham, MA, USA) by enzyme-linked immunosorbent assay and confirmed using radioimmunoprecipitation as previously described (8). Since the decrease in ANC and elevations in AST and ALT have been noted with treatment with rituximab and methotrexate, we evaluated these values in patients on the ITI protocol. ANC, ALT, and AST levels were monitored bi-weekly during ITI followed by monthly monitoring until return to baseline levels. Flow cytometry was performed in CLIA certified laboratories to define the following cell populations: CD19, CD3, CD3CD4, CD3CD8. Lymphocyte quantitation including CD19% was evaluated to monitor B cell suppression and B cell reconstitution. Lymphocyte quantitation was performed every 4 weeks until B cell recovery, then every 3–6 months. B cell depletion was defined as detection of CD19% below 1%. B cell reconstitution was measured in terms of normalization of CD19% to the normal range for the age as previously described (40). Additionally, CD3, CD4, and CD8% were evaluated to monitor T cell response. Titers against Diphtheria, Tetanus, Pneumoccoal, Measles, Mumps, and Rubella (MMR) were assessed after vaccination and humoral response to vaccines were categorized as adequate (immune) or inadequate (not immune). Humoral response to Tetanus, Diphtheria, and MMR were determined to be adequate based on the respective CLIA certified laboratory reference ranges. The response to the Pneumoccocal vaccine was determined to be adequate if >50% of serotypes had an antibody concentration of >1.3 micrograms per milliliter, as all patients were <6 years old (44). B cell reconstitution, vaccination status at baseline and after ITI, and titers against routine vaccines were collected to assess if administration of rituximab resulted in long-term immunodeficiency. Data collection continued until October 2019 or until at least 6 months had passed since the initiation of ERT and ITI, at which time the database was locked for analysis.

### Statistics

Overall survival and ventilator-free survival for patients who received ITI were analyzed using the Kaplan-Meier method with two-tailed *P*-values generated using the log-rank test and compared to a historical cohort of CRIM-negative IPD patients who received ERT monotherapy (15). Age at ERT, age at diagnosis, longitudinal anti-rhGAA IgG antibody titers, and LVMI were compared using Wilcoxon/Kruskal-Wallis rank-sum test. Analyses were performed with JMP Pro 14.0. Descriptive data are presented as medians.

## Results

### Patients and Treatment Details

From our international cohort of infantile Pompe disease patients (IPD), 34 patients (25 CRIM-negative and 9 CRIM-positive) who met all inclusion criteria and had received a short-course of ITI with rituximab, methotrexate, and/or IVIG in ERT-naïve setting were identified. ADDIN EN.CITE (23) Of the 25 CRIM-negative patients, 17 patients (CN1 to CN17) were included in our previous publication and met the inclusion criteria for the current study. Three CRIM-negative IPD patients (CN1, CN2, and CN6) received ITI with rituximab and methotrexate and did not receive IVIG, as per the local treating physician's decision.

Patient demographics, age at diagnosis, age at ERT initiation, *GAA* variants, dose of ERT, current age, age at death, and CRIM status are shown in Table 1. The median age at diagnosis was 2.4 months (range, 0.0–5.9 months) and 4.2 months (range, 0.0–10.9 months) for CRIM-negative and CRIM-positive IPD groups, respectively (Table 2). The median age at ERT and ITI initiation was 3.1 months (range, 0.1–6.7 months) and 4.8 months (range, 0.1–11.0 months) for CRIM-negative and CRIM-positive groups, respectively (Table 2). There were no statistically significant differences in age at diagnosis (*p* = 0.3189) or age at ERT initiation (*p* = 0.2828) between CRIM-negative and CRIM-positive IPD patients. At the time of database lock, 27 IPD patients were alive (18 CRIM-negative and 9 CRIM-positive) and 7 CRIM-negative IPD patients were deceased (Table 1). No statistically significant differences were observed in age at diagnosis (*p* = 0.2584) or age at ERT initiation (*p* = 0.2246) between living and deceased IPD patients.

**Table 1:** Demographics and treatment history.

**Patient/Gender**	***GAA* disease-associated variants**		**CRIM status**	**Age at diagnosis (months)**	**Age at ERT initiation (months)**	**Current age (months)**	**ERT dose at the time of initiation**
	**Allele 1**	**Allele 2**					
**ALIVE PATIENTS**							
CN1/F	c.341insT	c.341insT	Negative	1.9	3.8	148.8	20 mg/kg EOW^*^
CN3/F	c.2608C>T	c.2608C>T	Negative	2.5	3.0	112.0	20 mg/kg EOW^*^
CN4/M	c.546+2T>C	c.546+2T>C	Negative	3.5	4.6	111.2	20 mg/kg EOW
CN5/F	c.236_246del	c.236_246del	Negative	2.0	2.5	108.4	20 mg/kg EOW^*^
CN10/M	c.2560C>T	c.1292_1295dupTGCA	Negative	2.4	2.6	92.3	20 mg/kg Weekly^*^
CN11/F	c.2560C>T	c.2560C>T	Negative	0.3	1.3	88.8	20 mg/kg EOW^*^
CN12/F	c.258dupC	c.2227C>T	Negative	2.6	3.1	71.1	20 mg/kg EOW^*^
CN13/M	c.1754+2T>A	c.1822C>T	Negative	0.9	1.8	87.5	20 mg/kg EOW^*^
CN14/F	c.2237G>A	c.437delT	Negative	5.9	6.6	71.5	20 mg/kg EOW
CN16/F	c.2560C>T	c.2560C>T	Negative	Prenatal	0.1	50.5	20 mg/kg EOW^*^
CN17/M	c.2560C>T	c.525delT	Negative	3.3	3.6	43.1	20 mg/kg Weekly^*^
CN18/F	c.1195-18_2190-20del	c.1195-18_2190-20del	Negative	3.9	4.4	53.7	20 mg/kg EOW^*^
CN19/M	c.1827C>G	c.2662G>T	Negative	0.3	0.8	41.5	20 mg/kg EOW^*^
CN20/M	c.525delT	c.1694_1697delTCTC	Negative	0.9	1.0	20.2	20 mg/kg EOW^*^
CN22/M	c1548G>A	c2560C>T	Negative	4.9	5.4	41.8	20 mg/kg EOW
CN23/M	c.525delT	c.2560C>T	Negative	0.7	1.4	39.0	20 mg/kg EOW^*^
CN24/M	c.1051delG	c.1579delA	Negative	0.4	0.5	27.0	20 mg/kg EOW^*^
CN25/M	c.525delT	c.2560C>T	Negative	Prenatal	0.1	8.1	40 mg/kg EOW
CP1/M	c.1912G>T	c.2481+102_2646+31del	Positive	4.5	4.8	70.6	20 mg/kg EOW^*^
CP2/M	c.2457_246delCTG	c.2560C>T	Positive	3.8	4.0	70.9	20 mg/kg EOW^*^
CP3/F	c.1844G>A	c.1844G>A	Positive	10.9	11.0	109.5	20 mg/kg EOW
CP4/M	c.2105G>T	c.2512C>T	Positive	Prenatal	0.7	73.1	20 mg/kg EOW
CP5/F	c.525delT	c.2481+110_2646+39del	Positive	6.0	7.3	87.8	20 mg/kg EOW
CP6/F	c.1841C>A	c2481+102_2646+31del	Positive	4.2	5.1	46.5	40 mg/kg EOW^*^
CP7/M	c.1118T>G	c.1118T>G	Positive	Prenatal	0.1	21.2	20 mg/kg Weekly^*^
CP8/M	c.716delT	c.871C>T	Positive	6.0	6.4	42.9	20 mg/kg EOW^*^
CP9/F	c.1843G>A	c.1933G>C	Positive	0.1	0.9	36.9	20 mg/kg EOW
**DECEASED PATIENTS**							
CN2/M	c.1548G>A	c.525delT	Negative	2.4	3.6	56.9	20 mg/kg EOW
CN6/F	c.525delT	c.2560C>T	Negative	0.3	0.4	63.2	20 mg/kg Weekly
CN7/F	c.2560C>T	c.2560C>T	Negative	3.0	3.4	25.4	20 mg/kg EOW
CN8/F	c.525_526delTG	c.525_526delTG	Negative	5.5	6.7	30.0	20 mg/kg EOW
CN9/F	c.2560C>T	c.2560C>T	Negative	3.2	3.9	15.0	20 mg/kg EOW
CN15/F	c.2560C>T	c.2560C>T	Negative	5.1	6.6	15.5	20 mg/kg EOW^*^
CN21/F	c.2238G>A	c.2560C>T	Negative	5.9	6.3	25.1	40 mg/kg EOW^*^

*GAA, gene encoding acid α-glucosidase; CRIM, Cross-reactive immunologic material; ERT, enzyme replacement therapy; EOW, every other week*.

* *Patient was initiated on or subsequently received ERT at a higher dose than the recommended dose of 20 mg/kg EOW*.

**Table 2:** Summary of age at diagnosis, age at ERT initiation, anti-rhGAA IgG antibody titer, B cell, and LVMI.

	***N***	**Median**	**Range**
**Age at diagnosis**			
CRIM-negative	25	2.4 months	0.0–5.9 months
• Alive CRIM-negative	18	2.0 months	0.0–5.9 months
• Deceased CRIM-negative	7	3.2 months	0.3–5.9 months
CRIM-positive (all alive)	9	4.2 months	0.0–10.9 months
Alive (CRIM-negative and CRIM-positive)	27	2.4 months	0.0–10.9 months
**Age at ERT initiation**			
CRIM-negative	25	3.1 months	0.1–6.7 months
• Alive CRIM-negative	18	2.5 months	0.1–6.6 months
• Deceased CRIM-negative	7	3.9 months	0.4–6.7 months
CRIM-positive	9	4.8 months	0.1–11.0 months
Alive (CRIM-negative and CRIM-positive)	27	3.0 months	0.1–11.0 months
**Peak anti-rhGAA IgG antibody titer**			
CRIM-negative	25	200	0–51,200
• CRIM-negative tolerized	21	0	0–6,400
• CRIM-negative nontolerized	4	38,400	25,600–51,200
CRIM-positive (all tolerized)	9	0	0–200
**Anti-rhGAA IgG antibody titer at last assessment (time since ERT initiation)**			
CRIM-negative	25	100 (108 weeks)	0–51,200 (19–351 weeks)
• CRIM-negative tolerized	21	0 (103 weeks)	0–6,400 (19–351 weeks)
• CRIM-negative nontolerized	4	19,200 (228 weeks)	3,200–51,200 (72–343 weeks)
CRIM-positive (all tolerized)	9	0 (104 weeks)	0–100 (35–272 weeks)
**B cell response**			
Time to B cell depletion	31	3 weeks	1–5 weeks
Time to B cell reconstitution	33	17 weeks	11–54 weeks
**LVMI at baseline**			
CRIM-negative	20	178.2 g/m^2^	55.5–448.9 g/m^2^
CRIM-positive	8	221.0 g/m^2^	93.98–628.6 g/m^2^
**LVMI at most recent follow-up (time since ERT initiation)**			
CRIM-negative	24	62.9 g/m^2^ (84 weeks)	46.0–257.0 g/m^2^ (9–437 weeks)
CRIM-positive	8	69.7 g/m^2^ (73 weeks)	61.0–174.6 g/m^2^ (23–102 weeks)

*N, number; CRIM, cross-reactive immunologic material; ERT, enzyme replacement therapy; LVMI, left ventricular mass index*.

### Anti-rhGAA IgG Antibody Titers (Table 3)

**Figure 1:**
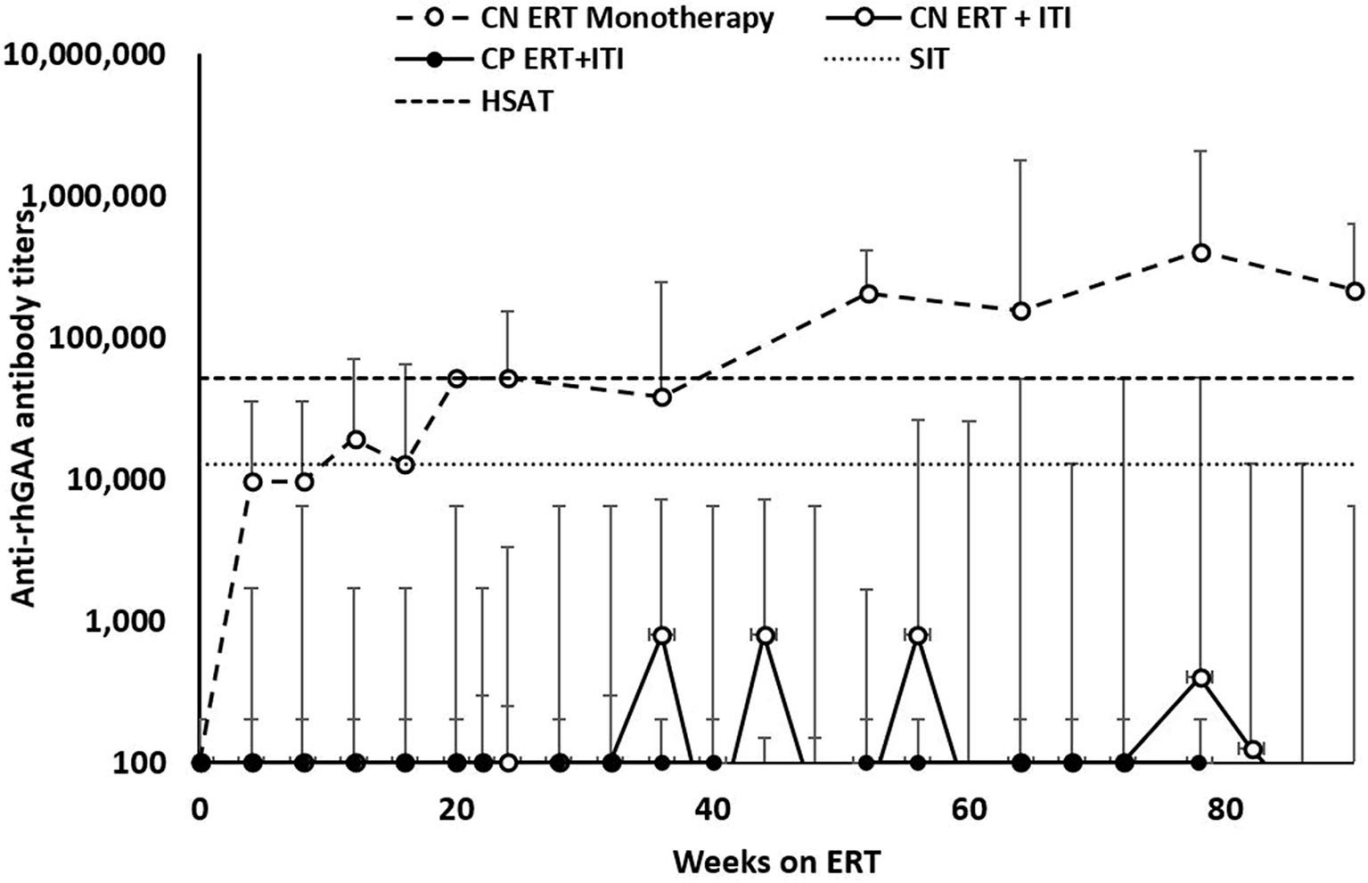
Longitudinal anti-rhGAA IgG antibody titers in IPD patients treated with immune tolerance induction. CRIM, cross-reactive immunologic material. CN, CRIM-negative; CP, CRIM-positive; ERT, enzyme replacement therapy; ITI, immune tolerance induction; SIT, sustained intermediate titer; HSAT, high and sustained antibody titer; rhGAA, recombinant human acid alpha-glucosidase.

Of the 34 IPD patients, 30 patients [88%; 21 CRIM-negative (84%) and 9 CRIM-positive (100%)] were immune tolerant with the longest follow-up of 348 weeks following ITI. Of these 30 IPD patients, sixteen (47%; 11 CRIM-negative and 5 CRIM-positive) remained seronegative (did not develop detectable anti-rhGAA IgG antibodies) and 14 (41%; 10 CRIM-negative and 4 CRIM-positive) developed low antibody titers (defined as titers of ≤6,400) throughout the course of ERT. Of the four CRIM-negative IPD patients who failed to tolerize, two (6%) (CN10 and CN12) developed sustained intermediate titers (titers of ≥12,800 and <51,200) and two (6%) (CN13 and CN21) developed high and sustained antibody titers (titers of ≥51,200 on two or more occasions). None of the CRIM-positive IPD patients who received immunomodulation developed SIT or HSAT. There was no recognizable difference in baseline characteristics between CRIM-negative IPD patients who developed HSAT/SIT and those who maintained low/negative anti-rhGAA IgG antibody titers (Table 1). The median peak anti-rhGAA IgG antibody titers were 200 (range, 0–51,200) and 0 (range, 0–200) for ITI treated CRIM-negative and CRIM-positive IPD groups, respectively (Table 2). The median peak anti-rhGAA IgG antibody titers were 0 (range, 0–6,400) for tolerized (*n* = 21) and 38,400 (range, 25,600–51,200) for nontolerized CRIM-negative IPD patients (*n* = 4). The median anti-rhGAA IgG antibody titers at the final assessment were 100 (range, 0–51,200) for ITI treated CRIM-negative IPD and 0 (range, 0–100) for ITI treated CRIM-positive IPD at the median timepoint following ERT initiation of 108 weeks (range, 19–351 weeks) and 104 weeks (range, 35–272 weeks) for ITI treated CRIM-negative and CRIM-positive groups, respectively (Figure 1). The median anti-rhGAA IgG antibody titers at the final assessment were 0 (*n* = 21; range, 0–6,400) and 19,200 (*n* = 4; range, 3,200–51,200) for tolerized and nontolerized CRIM-negative IPD patients, respectively. Overall, 88% of IPD patients who received immunomodulation in the ERT-naïve setting either remained seronegative or maintained low anti-rhGAA IgG antibody titers (Table 3).

**Table 3:** B cell reconstitution, infections, vaccination, anti-rhGAA IgG antibody titers, and ITI protocol deviations in IPD patients treated with immunomodulation.

**ID**	**Rounds of ITI**	**B Cell recovery (weeks post-RTX)**	**Weeks post-RTX at last CD19% follow-up**	**Infections**	**Vaccination prior to ERT**	**Vaccination up to date for age**	**Anti-rhGAA IgG antibody titers**			**ITI protocol deviations**
							**Peak titers (weeks on ERT)**	**Last titers (weeks on ERT)**	**Immune tolerant (Yes/No)**	
**ALIVE PATIENTS**										
CN1	1	Yes (17)	17	No	NA	Yes	1,600 (38)	200 (103)	Yes	Did not receive IVIG
CN3	1	Yes (19)	43	*Enterococcus faecalis, Pseudomonas fluorescens/putida, Enterococcus raffinosus*	NA	Yes	0	0 (281)	Yes	IVIG: 1 dose during ITI + 2 doses after ITI
CN4	1	Yes (17)	21	*RSV infection*	NA	Yes	0	0 (284)	Yes	IVIG started at Week 4 on ERT
CN5	1	Yes (11)	73	No	NA	Yes	0	0 (269)	Yes	None
CN10	2	Yes (19)	195	No	NA	Yes	25,600 (198)	25,600 (198)	No	Maintenance rituximab every 2 to 3 months following ERT+ITI for 32 months
CN11	1	Yes (32)	156	No	NA	Yes	200 (69)	0 (351)	Yes	3rd cycle of MTX was administered with 4th ERT infusion instead of 3rd ERT infusion
CN12	1	Yes (25)	33	*Klebsiella pneumoniae*	NA	Yes	25,600 (94)	3,200 (258)	No	None
CN13	>2	Yes (17)	208	No	Yes	Yes	51,200 (71)	12,800 (343)	No	Multiple cycle of ITI
CN14	1	Yes (25)	82	*Aspiration pneumonia, enterovirus/rhinovirus*	Yes	Yes	200 (81)	200 (81)	Yes	None
CN16	1	Yes (27)	91	No	No	Yes	0	0 (174)	Yes	None
CN17	1	Yes (13)	29	No	NA	Yes	6,400 (54)	3,200 (135)	Yes	None
CN18	1	Yes (13)	45	*URI rhinorrhea; UTI E. Coli; Ear infection*	Yes	Yes	200 (34)	100 (161)	Yes	None
CN19	1	Yes (17)	159	NA	Yes	Yes	0	0 (112)	Yes	None
CN20	1	Yes (13)	13	NA	Yes	No	0	0 (19)	Yes	None
CN22	1	Yes (16)	29	No	No	No	200 (108)	200 (108)	Yes	None
CN23	1	Yes (20)	102	No	Yes	Yes	0	0 (144)	Yes	None
CN24	1	Yes (12)	36	No	NA	NA	0	0 (31)	Yes	None
CN25	1	Yes (19)	19	No	No	No	0	0 (31)	Yes	None
CP1	1	Yes (18)	21	NA	Yes	NA	0	0 (35)	Yes	None
CP2	1	Yes (13)	248	NA	Yes	Yes	0	0 (171)	Yes	None
CP3	1	Yes (43)	75	NA	NA	NA	100 (4)	100 (78)	Yes	None
CP4	1	Yes (19)	19	NA	No	Yes	200 (22)	0 (272)	Yes	None
CP5	1	Yes (13)	53	NA	Yes	Yes	0	0 (39)	Yes	None
CP6	1	Yes (14)	164	NA	NA	Yes	0	0 (117)	Yes	2nd cycle of MTX was withheld. Three cycles of MTX administered with 1st, 3rd, and 4th ERT infusions.
CP7	1	Yes (15)	37	No	No	Yes	100 (66)	100 (66)	Yes	None
CP8	1	Yes (17)	141	NA	NA	Yes	0	0 (105)	Yes	None
CP9	1	Yes (12)	28	NA	Yes	Yes	200 (26)	100 (104)	Yes	None
**DECEASED PATIENTS**										
CN2	1	Yes (23)	51	No	NA	Yes	0	0 (67)	Yes	Two doses of MTX with 3rd ERT infusion and additional cycles of MTX administered with 4th, 5th, and 6th ERT infusions. Did not receive IVIG.
CN6	1	NA	NA	No	No	Yes	0	0 (70)	Yes	14 cycles of MTX administered with first 14 ERT infusions (Total 42 doses). Did not receive IVIG.
CN7	2	Yes (55)	59	No	Yes	Yes	6,400 (31)	3,200 (91)	Yes	Received a second cycle of ITI
CN8	2	Yes (43)	43	No	NA	Yes	6,400 (29)	6,400 (46)	Yes	Received a second cycle of ITI
CN9	1	Yes (22)	35	No	NA	Yes	1,600 (38)	800 (45)	Yes	IVIG initiated at Week 4 on ERT
CN15	1	Yes (22)	31	No	Yes	No	200 (3)	200 (32)	Yes	None
CN21	1	Yes (13)	17	NA	Yes	Yes	51,200 (65)	51,200 (72)	No	None

*RTX, rituximab; ERT, enzyme replacement therapy; NA, not available; wks, weeks; ITI, immune tolerance induction; MTX, methotrexate; IVIG, intravenous immunoglobulin*.

### Safety Measures

#### ANC, ALT, and AST Data

In the first 10 weeks on ERT and ITI, AST and ALT data were available for 28 IPD patients. Only one IPD patient (CN14) had an increase in AST >3 times baseline value and subsequently decreased to baseline levels 6 weeks following the last dose of rituximab. None of the other IPD patients exhibited such an increase in AST or ALT during immunomodulation. AST decreased to baseline levels 6 weeks following the last dose of rituximab. Moreover, since methotrexate and rituximab can induce neutropenia, ANC data for the first 10 weeks on ITI were analyzed and were available in 21 IPD patients. Eight patients (6 CRIM-negative and two CRIM-positive; patients: CN10, CN11, CN12, CN13, CN16, CN24, CP6, and CP7) developed ANCs of <750 cells/mm^3^ following immunomodulation. Neutropenia was transient and ANC level returned to normal in all patients within 23 weeks following cessation of immunomodulation.

#### Infections During Immunomodulation

Detailed information on the presence or absence of infections during ITI was available for 23 IPD patients (Table 3): five patients (CN3, CN4, CN12, CN14, and CN18) experienced infections while immune suppressed (23). Of these five IPD patients, details on four IPD patients have been reported previously (23). Central line infections and bacteremia were observed in Patient CN3 (Enterococcus faecalis, Pseudomonas fluorescens/putida, and Enterococcus raffinosus) and Patient CN12 (Klebsiella pneumoniae) requiring central line removal and antibiotic treatment. Patient CN4 had a respiratory syncytial virus infection and Patient CN14 suffered an episode of aspiration pneumonia and enterovirus/rhinovirus infection during immunomodulation. Patient CN18 experienced rhinorrhea, ear infection, and Escherichia coli urinary tract infection 4 weeks following completion of rituximab administration. Infections were managed with antibiotics without interrupting ERT infusions or ITI therapy. Overall, ITI was safely tolerated without any life-threatening infections.

### B Cell Reconstitution

Longitudinal follow-up of CD19% was available for 33 IPD patients (except patient CN6), with the longest follow-up of 248 weeks following the last dose of rituximab (Table 3). T cell percentages (CD3+, CD4+, CD8+) were within normal ranges for age making CD19% an appropriate measure for B cell reconstitution (Supplementary Figure 2). Complete B cell reconstitution, defined as normalization of CD19% for age, was seen in all 33 IPD patients. The median time to B cell depletion was 3 weeks (*n* = 31; range, 1–5 weeks) following initiation of ERT + ITI and the median time to complete B cell reconstitution was 17 weeks (*n* = 33; range, 11–54 weeks) following the last dose of rituximab. Following B cell reconstitution, all patients continued to maintain normal B cell counts, as measured by CD19%, with a median follow-up of 43 weeks (*n* = 33; range, 11–248 week). B cell reconstitution within 3 months following the last dose of rituximab was observed in three IPD patients (CN5, CN24, and CP9) and three patients (CN7, CN8, and CP3) experienced B cell recovery later than 9 months following the last dose of rituximab.

### Vaccination and Titers Against Vaccines

Data on routine vaccination prior to initiation of ITI were available on 19 IPD patients (13 CRIM-negative and 6 CRIM-positive) (Table 3). Of these 19 patients, 13 patients received age-appropriate vaccination prior to immunomodulation. Vaccination details post immunomodulation were available on 31 IPD patients (24 CRIM-negative and 7 CRIM-positive) and all except four CRIM-negative patients (CN15, CN20, CN22, and CN25) were up to date on their routine vaccination. Patient CN22 did not receive any vaccinations as per the decision of the parents/legal guardians. Patient CN15 was deceased, due to disease progression, prior to completion of routine immunization. Immunization was not yet resumed in Patients CN20 and CN25 at the time of database lock. After B cell reconstitution, titers against routine vaccines were performed in 12 patients (8 CRIM-negative and 4 CRIM-positive) and were categorized as adequate or inadequate based on the reference antibody values (Table 4). Two CRIM-positive IPD patients (CP2 and CP8) demonstrated an inadequate response to certain serotypes of the Pneumococcal vaccine. These two CRIM-positive IPD patients (CP2 and CP8) had an adequate humoral response to other vaccines where titers against the vaccine were performed. Four IPD patients (CN18, CN19, CN21, and CP2) had received age-appropriate vaccination prior to ITI and had titers against vaccines available (Tables 3, 4). All four IPD patients showed adequate humoral responses, although, it was not possible to determine if the response to the pre-ITI vaccine was maintained or lost, as all four patients received revaccination following complete B cell reconstitution after cessation of immunomodulation.

**Table 4:** Humoral response to routine vaccinations.

**Patient**	**Tetanus**	**Diphtheria**	**Measles**	**Mump**	**Rubella**	**Pneumoccocal**
CN3	Adequate	ND	Adequate	Adequate	Adequate	Adequate
CN11	Adequate	Adequate	Adequate	Adequate	Adequate	ND
CN12	Adequate	Adequate	Adequate	Adequate	Adequate	Adequate
CN16	Adequate	Adequate	ND	ND	ND	ND
CN17	Adequate	Adequate	Adequate	Adequate	Adequate	Adequate
CN18	Adequate	Adequate	Adequate	Adequate	ND	ND
CN19	Adequate	Adequate	ND	Adequate	Adequate	ND
CN21	Adequate	Adequate	ND	ND	ND	ND
CP2	Adequate	ND	Adequate	Adequate	Adequate	Inadequate
CP6	Adequate	Adequate	ND	ND	Adequate	ND
CP8	Adequate	Adequate	ND	ND	Adequate	Inadequate
CP9	ND	ND	ND	ND	ND	Adequate

*ND, Not done*.

### Left Ventricular Mass Index (LVMI)

LVMI at baseline was available for 28 IPD patients (20 CRIM-negative and 8 CRIM-positive) and at follow-up for 32 IPD patients (24 CRIM-negative and 8 CRIM-positive) (Table 5). Median LVMI at baseline was 178.2 g/m^2^ (*n* = 20; range, 55.5–448.9 g/m^2^) and 221.0 g/m^2^ (*n* = 8; range, 93.98–628.6 g/m^2^) for CRIM-negative and CRIM-positive IPD groups, respectively, with the upper limit of normal LVMI at 64 g/m^2^. Median LVMI at the most recent follow-up of CRIM-negative IPD patients was 62.9 g/m^2^ (*n* = 24; range, 46.0–257.0 g/m^2^) at a median time since ERT initiation of 84 weeks (range, 9–437 weeks). Median LVMI at the most recent follow-up of CRIM-positive IPD patients was 69.7 g/m^2^ (*n* = 8; range, 61.0–174.6 g/m^2^) at a median time since ERT initiation of 73 weeks (range, 23–102 weeks). It is important to note that all patients experienced decreases in their LVMI with 17 IPD patients (13 CRIM-negative and 4 CRIM-positive) having LVMIs within the normal range (below 64 g/m^2^) at the most recent follow-up. In contrast, CRIM-negative IPD patients from the original alglucosidase alfa clinical trials, who were not tolerized, but treated with ERT monotherapy, had progressive increases in their LVMI beyond the first 6 months on ERT (8, 9, 45, 46).

**Table 5:** Efficacy of ERT + ITI.

**ID**	**LVMI**		**Motor Status**		**Ventilation Status**		**Feeding Status**	
	**Baseline**	**Final assessment (weeks on ERT)**	**Baseline**	**Final assessment (weeks on ERT)**	**Baseline**	**Final assessment (weeks on ERT)**	**Baseline**	**Final assessment (weeks on ERT)**
**ALIVE PATIENTS**								
CN1	NA	NA	Hypotonia	Ambulatory (378)	No support	No support (378)	Oral	Oral (378)
CN3	160.3	57.8 (437)	Head lag, severe hypotonia, and motor delay	Ambulatory; wheelchair as needed, mostly for transportation (450)	Oxygen	No support (450)	NG tube	Oral (450)
CN4	445.8	68 (274)	Head lag, and antigravity movements (arms> legs)	Can move arms against gravity (286)	Oxygen and BiPAP at night	Invasively ventilated (271)	NG tube	G Tube (271)
CN5	277	80 (334)	Severe hypotonia, floppy baby, and no head or neck control	Ambulatory (76)	Oxygen	No support (76)	NG tube	Oral (76)
CN10	NA	58.4 (227)	Hypotonia	Ambulatory (199)	Invasively ventilated	No support (199)	TP	Oral (199)
CN11	140.6	53.7 (252)	Motor status and milestones appropriate for the age	Ambulatory (182)	No support	No support (213)	Oral	CPAP with nasal mask (199)
CN12	156.7	53.82 (208)	Hypotonia	Ambulatory (273)	No support	Vest/cough assist (273)	Oral	Oral (273)
CN13	NA	53.5 (217)	Hypotonia	Severely hypotonic, unable to hold head up, rollover, or sit unassisted. Can move both arms weakly (350)	No support	Invasively ventilated (350)	Oral	G Tube (350)
CN14	176	48 (105)	Hypotonia	NA	BiPAP	BiPAP (92)	G tube	G tube (92)
CN16	65.4	58.8 (48)	Head lag, and hypotonia	Normal developmental milestones (58)	CPAP for a week	No support (37)	Oral	Oral (37)
CN17	433.1	49.9 (124)	Normal symmetric bulk and appeared to have normal tone	Stands with support and braces, sitsunassisted rollsside to side, and can lift head up. Crawls and pushes to quadruped and creeps on hands and knees. (130)	No support	No support (130)	Oral	G Tube, eating puree orally (130)
CN18	448.9	62.7 (185)	Delayed motor milestones	Ambulatory (195)	Nasal O2	Recommended CPAP at night (195)	Oral and NG tube	Oral (195)
CN19	NA	63.02 (163)	NA	Ambulatory (162)	NA	No support (162)	NA	Oral (162)
CN20	180.4	80.4 (23)	Hypotonia	Generalized hypotonia (50)	No support	Overnight BiPAP (59)	NG tube	G tube (59)
CN22	211.9	192.5 (26)	Mild hypotonia and delayed head control at 3 months but rolling	Ambulatory. Walks, runs, jumps, feeds self, plays with siblings, dresses self, and walks upstairs (136)	No support	No support (136)	NG tube	NG tube and oral (136)
CN23	156.2	60.2 (141)	Mild hypotonia	Ambulatory; meeting developmental milestones (101)	No support	No support (101)	Oral	Oral (101)
CN24	158	46 (47)	Delayed milestones	Ambulatory. Walking, running, and jumping (88)	No support	No support (156)	Oral	Oral (156)
CN25	55.5	62.3 (9)	Age appropriate gross motor skill development	Meeting developmental milestones (27)	No support	No support (27)	Oral	Oral (27)
CP1	423.6	75.1 (31)	Mild axial hypotonia with head lag	Ambulatory with moderate hypotonia. Severe delay in gross motor development (196)	No support	No support (220)	Oral, NG Tube at night	Oral (220)
CP2	186	61 (81)	Good muscle strength and tone. Minimal head lag, lifts head up	Not ambulatory. Does not move legs in supine, requires assistance for head control in supported sitting (274)	No support	Invasively ventilated (274)	NG Tube	G Tube (274)
CP3	628.6	174.6 (76)	Head lag.	Ambulatory. Mild proximal weakness (100)	No support	No support (411)	Oral	Oral (411)
CP4	251.7	75.6 (23)	NA	Ambulatory. Uses a wheelchair for transportation (297)	No support	Oxygen at night (268)	Oral	Oral (297)
CP5	248	105.3 (98)	NA	NA	NA	NA	NA	NA
CP6	NA	NA	General hypotonia	Ambulatory. Low muscle tone, global muscle weakness, and delayed motor skills (167)	High flow nasal cannula	No support (167)	NG Tube	Oral (167)
CP7	93.98	64.2 (37)	Normal muscle tone	Ambulatory; Low tone (72)	No support	No support (72)	Oral	Oral (72)
CP8	194	62 (70)	Rolling supine to left and right, and side lying	Ambulatory (137)	No support	No support (137)	Oral	Oral (137)
CP9	122	63 (102)	Slightly decreased tone. Able to control head without support	Ambulatory. Able to get up and down from the floor and steps with assistance (130)	No support	No support (135)	Oral	Oral (135)
**DECEASED PATIENTS**								
CN2	NA	257 (63)	Hypotonia	Prop-sits unassisted, rolls from supine to side lying, and bears weight through lower extremities in supported standing (80)	Transient ventilation for 3 days	No support (67)	Oral	Oral (80)
CN6	409.6	92.3 (53)	Axial hypotonia, withdraws extremities to stimulation, weak grasp	Sits with support and minimal capacity for weight-bearing on lower extremities (53)	Invasively ventilated	Invasively ventilated (off ventilator 10–12 hours a day) (58)	G tube	G tube (58)
CN7	317.2	144.9 (54)	Head lag and unable to sit or rollover	Standing with support (46)	Invasively ventilated	Oxygen and BiPAP at night (46)	NJ Tube	G Tube (46)
CN8	347.1	107.9 (36)	Severe hypotonia	Able to move arms against gravity but near-complete lower extremity immobility (50)	Invasively ventilated	BiPAP at night (50)	NG Tube	G Tube (50)
CN9	220	83 (39)	Unable to independently hold head or sit unsupported	Not able to hold head or sit unsupported (46)	No support	Invasively ventilated (46)	NG Tube	GJ Tube (46)
CN15	127.5	118.3 (28)	Hypotonia	Not ambulatory (38)	Invasively ventilated	Invasively ventilated (38)	NG tube	G tube (38)
CN21	160.1	76 (15)	NA	Not ambulatory, severely limited motor skills (56)	Invasively ventilated	Invasively ventilated (56)	NG Tube	G Tube (56)

*LVMI, left ventricular mass index; ERT, enzyme replacement therapy; ITI, immune tolerance induction; NA, not available; NG, nasogastric tube; TP, transpyloric*.

*Two CRIM-positive IPD patients had inadequate humoral response to Pneumoccocal vaccine while demonstrating adequate response to other age-appropriate vaccines. It is not clear if the lack of response to Pneumoccocal vaccine in these two patients was due to immunosuppression or normal variability in the efficacy of vaccine. As per the CDC, the efficacy of PCV13 is 45.6–75.0% and PPSV23 is 60–70%*.

### Overall and Invasive Ventilator-Free Survival (Figure 2)

Of the 34 IPD patients, 27 (18 CRIM-negative and all 9 CRIM-positive) were alive and seven were deceased (all CRIM-negative; CN2, CN6, CN7, CN8, CN9, CN15, and CN21) at the time of database lock. Among the living IPD patients, the median current age is 70.6 months (range, 8.1–148.8 months). Of the seven deceased CRIM-negative patients, the median age of death was 25.4 months (range, 15.0–63.2 months). The cause of death for all seven was cardiorespiratory failure due to disease progression and was unrelated to ITI. However, there were no statistically significant differences in either age at diagnosis (*p* = 0.0896) or age at ERT initiation (*p* = 0.0693) between living and deceased CRIM-negative IPD patients (Table 1). Mortality in this population better reflects the extent of pathology prior to treatment initiation.

**Figure 2:**
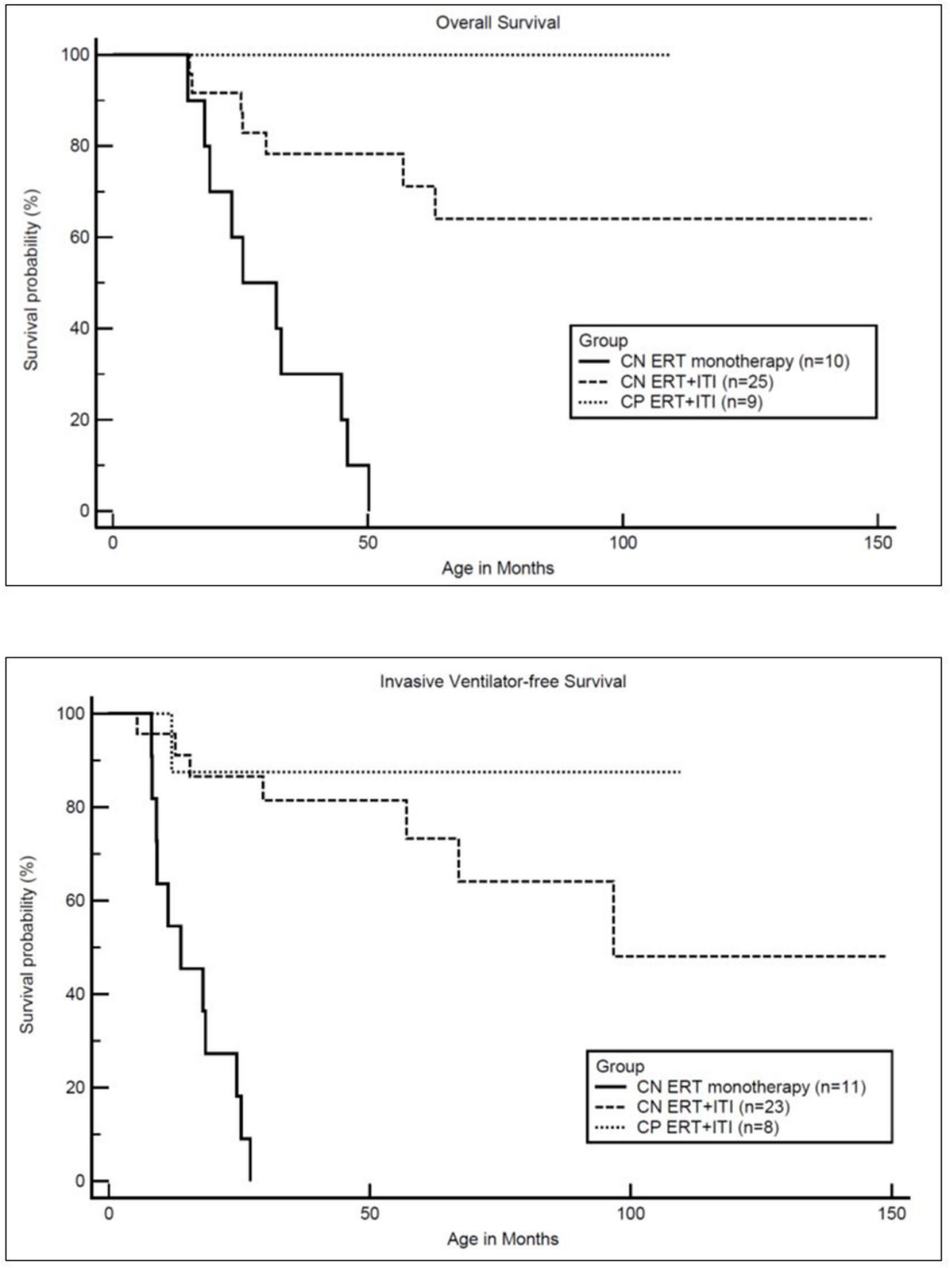
Kaplan-Meler survival analysis: overall and invasive ventilator-free survival. CN, CRIM-negative; CP, CRIM-positive; ERT, enzyme replacement therapy; ITI, immune tolerance induction.

Median age at ERT initiation was 3.0 months (range, 0.1–11.0 months) and 3.9 months (range, 0.4–6.7 months) and median age at diagnosis was 2.4 months (range, 0.0–10.9 months) and 3.2 months (range, 0.3–5.9 months) for living and deceased groups, respectively. Among living CRIM-negative IPD patients (*n* = 18) median age at diagnosis (2.0 months; range, 0.0–5.9 months) and median age at ERT initiation (2.5 months; range, 0.1–6.6 months) were earlier compared to the median age at diagnosis (3.2 months; range, 0.3–5.9 months) and median age at ERT initiation (3.9 months; range 0.4–6.7 months) in deceased CRIM-negative IPD patients (*n* = 7). Although lacking statistical significance, even a relatively short delay in ERT may impact extent of clinical benifits and lead to permanent muscle loss.

As previously reported, all CRIM-negative IPD patients from original clinical trials, who received ERT monotherapy, were either deceased or invasive ventilator-dependent by 27.1 months of age (17). In the current cohort of CRIM-negative IPD patients, 16 CRIM-negative IPD patients (64%) were living without the need for invasive ventilation with the age of the oldest survivor being 148.8 months (range, 8.1–148.8 months) (Figure 2). Invasive ventilator-free survival was significantly (*p* = 0.0010) improved in CRIM-negative IPD patients who received ERT with ITI compared to ERT monotherapy.

In the current analysis, at baseline, eleven CRIM-negative (CN1, CN9, CN11, CN12, CN13, CN17, CN20, CN22, CN23, CN24, and CN25) and seven CRIM-positive (CP1, CP2, CP3, CP4, CP7, CP8, and CP9) IPD patients did not require any respiratory support whereas six CRIM-negative IPD patients (CN2, CN6, CN7, CN8, CN15, and CN21) were invasively ventilated (Table 5). Of these six invasively ventilated IPD patients, three (CN7, CN8, and CN10) were able to come off invasive ventilation with patient CN10 requiring no respiratory support at the most recent follow-up. At the most recent follow-up, 13 CRIM-negative (CN1, CN2, CN3, CN5, CN10, CN11, CN16, CN17, CN19, CN22, CN23, CN24, and CN25) and 6 CRIM-positive (CP1, CP3, CP6, CP7, CP8, and CP9) IPD patients did not require respiratory assistance whereas six CRIM-negative (CN4, CN6, CN9, CN13, CN15, CN21) and one CRIM-positive (CP2) IPD patients were invasively ventilated.

## Discussion

The negative impact of high and sustained anti-rhGAA IgG antibody titers to treatment response has been evident since the first clinical trial of alglucosidase alfa (46). Although the published literature has supported that abrogation of the immune response to ERT improves the efficacy of ERT in patients with Pompe disease (22, 23, 47), the long-term safety with the use of rituximab in such a young and medically fragile population has been an outstanding concern, thus prompting this study. This is the largest cohort of CRIM-negative and CRIM-positive IPD patients, to our knowledge, treated with ITI in the ERT-naïve setting with the longest follow-up of 148 months on ERT. It is also the youngest cohort of patients that have received rituximab for any indication; 16 patients were initiated on rituximab ages ≤3 months. In the published literature, the experience on safety of rituximab has been reported on patinets aged 4 months to 18 years (33–35, 38, 48–53).

We found important clinical improvements from the initiation of a short course of ITI with rituximab, methotrexate, and IVIG concomitant with ERT. These improvements included reduced need for mechanical ventilation, LVMI, improved motor ability and longer overall survival. Importantly, three CRIM-negative IPD patients, who were invasively ventilated at baseline, no longer required invasive ventilation at the most recent follow-up, demonstrating a significant reversal in the disease course. This was an important finding as IPD patients are rarely able to come off ventilatory support once invasively ventilated. This further demonstrates the benefits of initiation of a short course of ITI with rituximab, methotrexate, and IVIG concomitant with ERT.

Immunomodulation was largely successful in reducing the development of anti-rhGAA IgG antibody titers. Thirty patients with IPD (88%), who received prophylactic ITI, either did not develop (*n* = 16) or maintained low anti-rhGAA IgG antibody titers (*n* = 14). Four CRIM-negative IPD patients developed anti-rhGAA IgG antibody titers in the SIT or HSAT ranges; one of the SIT patient's rhGAA IgG antibody titers subsequently decreased to 3,200 at the final assessment. There were no recognizable differences in baseline characteristics between IPD patients who maintained low antibody titers and IPD patients who developed SIT or HSAT. There is no apparent explanation as to why these four IPD patients did not respond to ITI similarly to other patients in the current cohort. One hypothesis for the lack of response is resistance to rituximab. Rituximab resistance is known to be a common occurrence in naïve patients; however, its mechanism is incompletely understood. The potential mechanism of rituximab resistance is Fc receptor genetic variants affecting the affinity of effector cells for rituximab, complement depletion, and loss or decreased expression of CD20 on target antigen (54). Another possible reason for the development of high sustained anti-rhGAA IgG antibody titers in a few cases (CN10, CN12, CN13, and CN21) is incomplete B cell depletion. Rituximab has important limitations in that it doesn't deplete plasma cells, as they do not express CD20. Additionally, murine models have shown that 5% of B cells in lymph nodes survive CD20 depletion strategies (55). Although another recognized challenge with the use of rituximab is infusion-related reactions; with an incidence of infusion-related reactions of 25% of NHL patients and 25% of CLL patients interestingly, we did not observe any infusion-related reactions to rituximab in the current cohort of IPD patients.

Major concerns with the use of rituximab in patients with neoplastic and autoimmune disorders consist of significant delays in B cell recovery, skewing of B cell subpopulation to immature phenotype, and inability to mount a protective humoral response to vaccines (32). The addition of methotrexate to rituximab in the immunosuppressive regimen IPD patients receive only heightens the potential concerns. Much of the published data on rituximab originated from the treatment of adult populations where the extent of symptomatic hypogammaglobulinemia with an average of 4–6% of patients on rituximab requiring IVIG for symptomatic hypogammaglobulinemia. Persistent hypogammaglobulinemia was seen in up to 40% of patients on rituximab (usually those on long courses of rituximab to treat lymphoma), vaccine response was sometimes altered, and B cell reconstitution could take up to 24 months (36, 37). Small studies in pediatric populations have been more optimistic showing normalization of B cells by 1 year in nearly all patients, regardless of indication or duration of rituximab, and hypogammaglobulinemia rates at a maximum of 22% (33, 34, 38, 52). Our cohort of IPD patients is the largest group of patients under 1 year of age evaluated for rituximab impact. Most notably, all of the IPD patients experienced complete B cell reconstitution after discontinuation of rituximab with a relatively fast median time to reconstitution (median = 17 weeks) supporting the contention that the younger the patient the more rapid the reconstitution after immunomodulation with rituximab.

Methotrexate is an inhibitor of dihydrofolate reductase, an enzyme necessary for the synthesis of purine nucleotides and thymidylate. It predominantly affects rapidly dividing cells, such as lymphocytes, by interfering with DNA synthesis and repair. In addition to impacting B and T cells, methotrexate can help to prevent the development of ADA to rituximab.Methotrexate's ability to prevent ADA development stems from different lines of evidence- A murine study showed that methotrexate's interaction with BAFF (B cell activating factor of the TNF family), a driver of B cell activation, is important in the prevention of ADA development (28, 29). In rheumatic disease, concomitant therapy with a biologic and methotrexate prevented the development of antibodies against the biologic (30). Available data on methotrexate's impact on B cells indicate that it does not impact the overall CD19% but rather impacts B cell subsets and immunoglobulin levels (56–58). Our tracking of B cell depletion is limited by inconsistent collection of absolute counts with consistent reporting of CD19%. Future analysis will be necessary to assess B cell subsets and immunoglobulin levels during immunomodulation and immune reconstitution.

Although data were available for a small number of patients (titers available for 12 patients), patients generally had protective vaccine titers to polysaccharide (T cell-independent) antigen and conjugated (T cell-dependent) antigen. All IPD patients demonstrated an adequate humoral response against tetanus and diphtheria vaccines, and all except two CRIM-positive IPD patients (CP2 and CP8) had adequate pneumococcal titers. It is not clear if the lack of response to polysaccharide vaccines in these two patients was due to immunosuppression or normal variability in response to pneumococcal vaccines.

The short course of prophylactic ITI was safely tolerated without any major adverse events. Although infections were reported in five CRIM-negative patients which required treatment with antibiotics and central line removal in two patients, no interruption in ERT or immunomodulation was required in any of the patients. At the most recent follow-up, seven IPD patients were deceased at a median age of 25.4 months (range, 15.0–63.2 months). The cause of death was cardiorespiratory failure due to disease progression and was unrelated to immunomodulation but likely pertained to the extent of disease progression prior to treatment. Overall survival was significantly (*p* = 0.0001) improved in CRIM-negative IPD patients who received ITI with ERT compared to CRIM-negative IPD patients on ERT monotherapy.

Various immunomodulation strategies using rituximab in patients with Pompe disease have been reported in the literature. The combination of rituximab, methotrexate, with or without IVIG initiated along with the first ERT infusion, as shown in the current study, has proven to be the most successful strategy in inducing immune tolerance in patients with IPD (25). The immunomodulation strategy reported by Elder et al. used a combination of rituximab, mycophenolate/sirolimus, and IVIG in five IPD patients (four CRIM-negative and one CRIM-positive) (27). This protocol required long-term immune suppression and more significantly delayed ERT initiation by at least 3 weeks which can be very detrimental in a rapidly progressive irreversible muscle disease (59). Poelman et al. utilized a combination of rituximab, methotrexate, and IVIG similar to our ITI protocol, however, with a different dosing schedule of methotrexate, in three IPD patients (one CRIM-negative and two CRIM-positive) (26). All three patients developed anti-rhGAA IgG antibodies with two developing HSAT. Although B cell reconstitution was observed in all three patients, B cell reconstitution also resulted in an increase in rhGAA IgG antibody titers. In contrast, our immunomodulation approach was able to tolerize 84% of CRIM-negative and 100% of CRIM-positive IPD patients as evidenced by the maintenance of low or complete absence of anti-rhGAA IgG antibody titers even well after B cell recovery (22, 23).

To our knowledge, this the largest cohort of patients with IPD treated with ITI in ERT-naïve settings and the largest cohort of pediatric patients under a year of age evaluated for the safety of rituximab. Overall, this short course of immune modulation in the ERT-naïve setting significantly increased the likelihood of achieving long-term immune tolerance to ERT and did not lead to any long-term sequelae. Patients who received ITI were able to receive routine vaccinations and demonstrated adequate humoral immune responses. The data suggest that short-course prophylactic immunomodulation with rituximab, methotrexate, and IVIG initiated in the ERT-naïve setting is safe and efficacious in achieving long-term immune tolerance to ERT. The addition of this ITI regimen to ERT is life-saving and our data show that the benefits of adding immune modulation (ITI regimen) outweigh the risks in this setting.

## Data Availability Statement

All datasets generated for this study are included in the article/Supplementary Material.

## Ethics Statement

The studies involving human participants were reviewed and approved by Duke Health Institutional Review Board. Written informed consent to participate in this study was provided by the participants' legal guardian/next of kin.

## Author Contributions

Conception and study design and development of methodology were performed by AD and PK. Analysis and interpretation of data were performed by AD, CB, JS, and PK. All authors contributed to the article and approved the submitted version.

## Conflict of Interest

AD has received research support from Sanofi Genzyme and Lysosomal Disease Network (LDN). CB has received research support from the National Institute of Health (NIH) T32 training grant (Award number AI007062-38). PK has received research/grant support from Sanofi Genzyme, Valerion Therapeutics, and Amicus Therapeutics, consulting fees and honoraria from Sanofi Genzyme, Amicus Therapeutics, Vertex Pharmaceuticals and Asklepios Biopharmaceutical, Inc. (AskBio), is a member of the Pompe and Gaucher Disease Registry Advisory Board for Sanofi Genzyme, Amicus Therapeutics, and Baebies, and has equity in Asklepios Biopharmaceutical, Inc. (AskBio), which is developing gene therapy for Pompe disease. The remaining authors declare that the research was conducted in the absence of any commercial or financial relationships that could be construed as a potential conflict of interest.

## Supplementary Material

Supplementary Figure 1 In patients with Pompe disease receiving ERT at dose of 20 or 40 mg/kg every other week, ITI with rituximab (4 weekly doses), methotrexate (3 cycles with first 3 ERT infusions; total 9 doses), and IVIG (every 4 weeks) is admintered as described in the figure. ^*^For patients receiving ERT at dose of 20 mg/kg or 40 mg/kg weekly, three cycle of methotrexate is administered with first three ERT infusion at weeks 0–2. The dosing of rituximab and IVIG remains the same.

Supplementary Figure 2 CD3 10th Percentile, CD4 10th Percentile, CD8 10th Percentile, and CD19 10th Percentile represent the lower limit of age-appropriate normal range for respective lymphocye subset.
