# Supplementary figures and images for: Benefits of Prophylactic Short-Course Immune Tolerance Induction in Patients With Infantile Pompe Disease: Demonstration of Long-Term Safety and Efficacy in an Expanded Cohort
Source: Front Immunol. 2020 Aug 6;11:1727. doi: 10.3389/fimmu.2020.01727 (PMC7424004; doi:10.3389/fimmu.2020.01727)

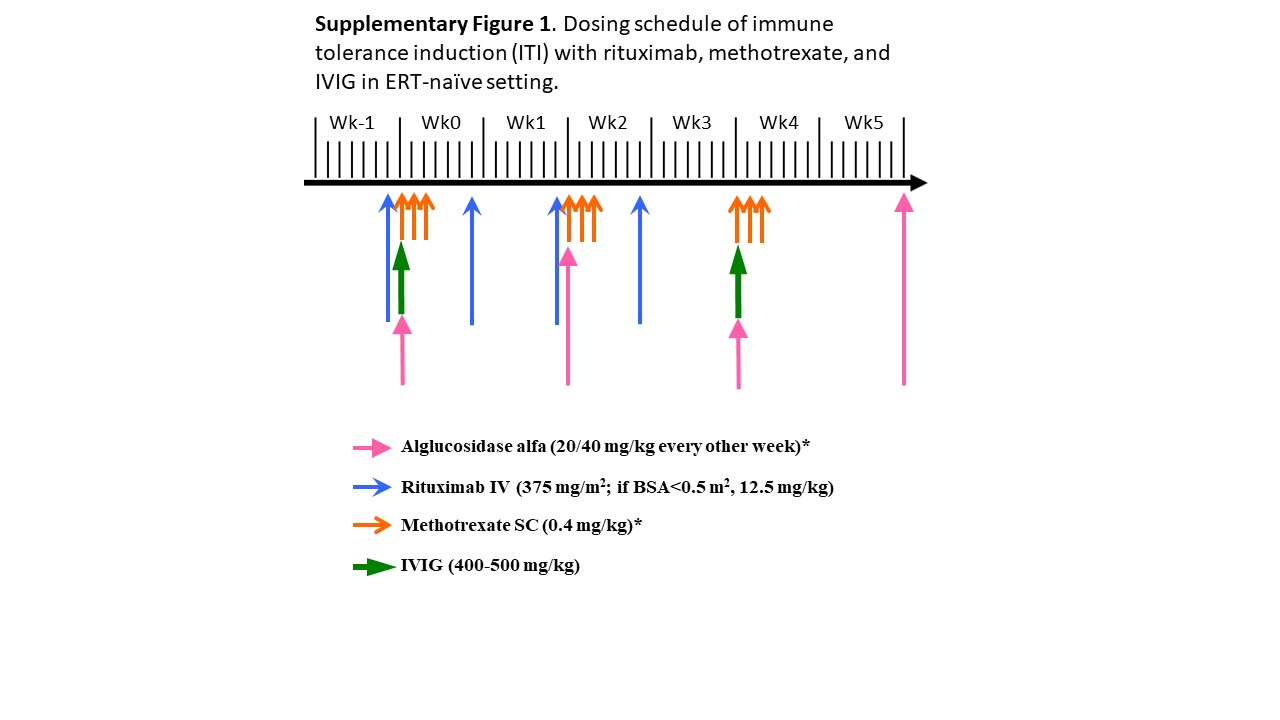

Supplement: Supplementary Figure 1 — In patients with Pompe disease receiving ERT at dose of 20 or 40 mg/kg every other week, ITI with rituximab (4 weekly doses), methotrexate (3 cycles with first 3 ERT infusions; total 9 doses), and IVIG (every 4 weeks) is admintered as described in the figure. *For patients receiving ERT at dose of 20 mg/kg or 40 mg/kg weekly, three cycle of methotrexate is administered with first three ERT infusion at weeks 0–2. The dosing of rituximab and IVIG remains the same. [file Image_1.jpg]

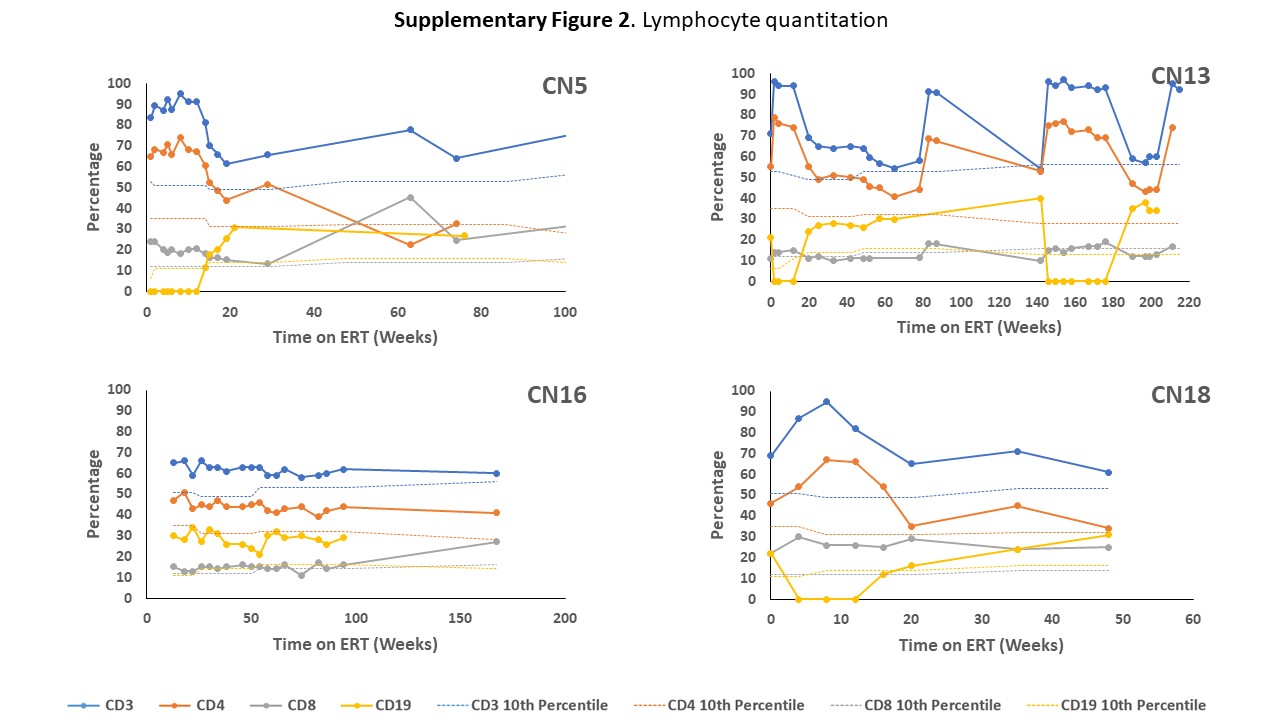

Supplement: Supplementary Figure 2 — CD3 10th Percentile, CD4 10th Percentile, CD8 10th Percentile, and CD19 10th Percentile represent the lower limit of age-appropriate normal range for respective lymphocye subset. [file Image_2.jpg]
